# NephroCheck as a Predictor of Acute Kidney Injury Following Coronary Artery Bypass Graft Surgery

**DOI:** 10.7759/cureus.75555

**Published:** 2024-12-11

**Authors:** Zain Khalpey, Pranav Jutla, Zacharya I Khalpey, Jessa Deckwa, Ujjawal Kumar

**Affiliations:** 1 Department of Cardiothoracic Surgery, HonorHealth, Scottsdale, USA; 2 Khalpey AI Lab, Applied & Translational AI Research Institute (ATARI), Scottsdale, USA; 3 School of Life Sciences, Arizona State University, Tempe, USA; 4 Department of Research, Nihon Kohden Digital Health Solutions, Irvine, USA; 5 School of Clinical Medicine, University of Cambridge, Cambridge, GBR

**Keywords:** acute kidney injury, biomarkers, cabg, cardiac surgery, igfbp7, nephrocheck, timp2

## Abstract

Background

Cardiac surgery-associated acute kidney injury (CSA-AKI) remains a significant complication following coronary artery bypass grafting (CABG), affecting 22%-30% of patients. This study evaluates the efficacy of NephroCheck, a biomarker-based test measuring insulin-like growth factor-binding protein 7 (IGFBP7) and tissue inhibitor of metalloproteinases-2 (TIMP2), in predicting postoperative AKI.

Methods

In this retrospective observational cohort study, 21 patients undergoing isolated CABG were analyzed. NephroCheck values were measured preoperatively, upon ICU admission, and at 24 hours post-admission. Patients with chronic kidney disease (CKD), heart failure, or preoperative serum creatinine of ≥2 mg/dL were excluded. AKI was defined using the Kidney Disease Improving Global Outcomes (KDIGO) criteria through postoperative day 7.

Results

Seven patients developed postoperative AKI. Preoperative NephroCheck values were significantly elevated in the AKI group compared to controls (1.34 (ng/ml)^2^/1000 ± 1.33 vs 0.54 (ng/ml)^2^/1000 ± 0.43, p = 0.009), while traditional renal function parameters showed no significant differences. Regression analysis identified preoperative NephroCheck as the only significant predictor of postoperative AKI (OR: 17.85, p = 0.047). Traditional risk factors, including preoperative renal function, Society of Thoracic Surgeons (STS) score, and operative times, showed no significant predictive value.

Conclusions

Preoperative NephroCheck testing demonstrates significant utility in predicting CSA-AKI following CABG, outperforming traditional risk assessment methods. These findings suggest that incorporating NephroCheck into routine preoperative assessments could enable more targeted preventive interventions, potentially reducing CSA-AKI incidence and its associated complications.

## Introduction

Acute kidney injury (AKI) remains a common and severe complication following cardiac surgery, affecting a substantial percentage of patients and significantly influencing both short- and long-term outcomes. Better known as cardiac surgery-associated acute kidney injury (CSA-AKI), this condition has an incidence rate ranging from 22% to 30% among patients undergoing procedures such as coronary artery bypass grafting (CABG) and valve surgeries, and it often leads to increased morbidity and mortality [1,2]. CSA-AKI has been identified as an independent predictor of poor prognostic outcomes, including increased risk of future cardiac events and progression to chronic kidney disease (CKD) [3]. Specifically, Hobson et al. state that even minimal elevations in serum creatinine (SCr) postoperatively are associated with increased mortality, highlighting the critical nature of this complication and the need for urgency to address it [3].

The pathophysiology of CSA-AKI is complex and multifaceted, with several mechanisms contributing to renal injury. During cardiac surgery, patients often experience periods of renal hypoperfusion, ischemia-reperfusion injury, and a systemic inflammatory response, all of which can adversely affect kidney function. The use of cardiopulmonary bypass (CPB), for example, can lead to significant fluctuations in blood flow and hemodynamic instability, causing renal tubular and endothelial damage. Additionally, systemic inflammation caused by surgical stress further exacerbates renal injury, making CSA-AKI a frequent and difficult-to-manage complication [4,5].

Zooming out, the financial burden of CSA-AKI is considerable, with a significant increase in hospitalization costs for patients who develop AKI following cardiac surgery. Research indicates that the average hospitalization cost for patients with CSA-AKI nearly doubles, trending around $77,000, compared to those without, adding substantial economic strain on healthcare systems [6]. This substantial cost difference is attributed to extended hospital stays, additional treatments, and the need for renal replacement therapy in severe cases. With the annual national cost increase related to CSA-AKI approaching $1 billion in the United States, there is yet another compelling need for effective prevention and early detection strategies [6].

One promising approach to early detection of CSA-AKI risk is through the use of biomarkers associated with early kidney stress and tubular injury. Two such biomarkers, insulin-like growth factor-binding protein 7 (IGFBP7) and tissue inhibitor of metalloproteinases-2 (TIMP2), have emerged as sensitive indicators of cellular stress in renal tubular cells [7]. These markers are associated with cell cycle arrest, which precedes the onset of AKI, allowing for the detection of kidney stress even before clinical signs of renal dysfunction become apparent [7,8]. The NephroCheck test serves as a diagnostic tool that measures urinary levels of IGFBP7 and TIMP2 to quantify early kidney stress, offering clinicians a valuable predictive measure of AKI risk within 12-48 hours before kidney injury becomes clinically detectable.

Research supports the use of the NephroCheck test as a reliable preoperative tool for identifying patients at high risk of CSA-AKI, particularly those undergoing complex and high-risk surgeries such as CABG. Studies have shown that elevated preoperative levels of IGFBP7 and TIMP2 correlate strongly with postoperative AKI [8], enabling more personalized perioperative care strategies.

This study aims to further evaluate the utility of the NephroCheck test as a prognostic tool for detecting AKI in patients undergoing CABG. Specifically, it seeks to assess the correlation between elevated preoperative and intraoperative NephroCheck values and the incidence of postoperative CSA-AKI within the critical 48-72-hour window postsurgery. Additionally, this study examines whether implementing targeted intraoperative resuscitation strategies in high-risk patients, as identified by NephroCheck, could influence postoperative outcomes. By incorporating NephroCheck into routine preoperative assessments, clinicians may enhance patient care through timely, personalized interventions, ultimately reducing CSA-AKI incidence and its associated clinical and economic burdens.

## Materials and methods

Patient inclusion and exclusion

This is a retrospective observational cohort study, comprised of patients who underwent isolated coronary artery bypass graft surgery via median sternotomy at Northwest Medical Center, Tucson, AZ, USA. All patients included were over the age of 18. Patients with CKD, diastolic or systolic heart failure, preoperative serum creatinine ≥ 2 mg/dL, or those who received methylene blue were excluded from the study.

Heart failure was defined using American Heart Association (AHA) criteria for diastolic heart failure as a left ventricular ejection fraction (LVEF) less than or equal to 40% by transthoracic echocardiography [9]. CKD was defined using an internationally conventional set of guidelines from the Kidney Disease: Improving Global Outcomes (KDIGO). These criteria define CKD as having a glomerular filtration rate (GFR) of less than 60 ml/min/1.73m2 and albuminuria with an albumin-creatinine ratio greater than 3 mg/mmol [10].

Data collection and analysis

Serial SCr levels were analyzed daily from the day of cardiac surgery (POD#0) until discharge. AKI was defined using SCr at POD#7 using KDIGO criteria shown in Table 1 [11]. Patient demographics, length of admission, and other parameters were recorded. Results were stored in a secure document. No patient identifiers were used. IRB approval (WIRB Study#1275376; IRB#20200195; February 5, 2020) and patient consent were obtained for relevant treatments and retrospective data interpretation. Data were summarized using descriptive statistics. For continuous variables, the mean and standard deviation (SD) were presented for normally distributed data, with nonparametric data presented as a median and interquartile range (IQR). Categorical variables were presented as N (%). All statistical analyses were performed using R v4.4.1 (R Foundation, Vienna, Austria) [12] and GraphPad Prism v10.1.0 for macOS (GraphPad Software, Boston, MA, USA) [13]. A p-value of less than 0.05 was considered significant as is conventional.

**Table 1:** KDIGO criteria for staging and classification of AKI KDIGO: Kidney Disease: Improving Global Outcomes; AKI: acute kidney injury; RRT: renal replacement therapy; eGFR: estimated glomerular filtration rate Our study used the KDIGO criteria [11] to identify patients who developed AKI by POD#7. KDIGO is an international organization that develops widely used clinical practice guidelines.

Stage	Serum creatinine	Urine output
1	1.5-1.9 x baseline; ≥ 0.3mg/dL increase	<0.5mL/kg/h for 6-12h
2	2.0-2.9 x baseline	<0.5mL/kg/h for ≥12h
3	3.0 x baseline; ≥ 4.0mg/dL increase; initiation of RRT; decrease in eGFR to ≤ 35mL/min/1.73m^2^	<0.3mL/kg/h for ≥24h

Clinical protocol

In protocol one, patients received three NephroCheck tests, first in the preoperative area, second on admission to the intensive care unit (ICU), and the third 24 hours following admission to the ICU. Due to missing values contributing to missing order sets or lack of lab staff, a second protocol was developed. In protocol two, patients received three NephroCheck tests first in the preoperative area, the second on postoperative day one, and the third on postoperative day two.

Ethical approvals

An institutional review board ethical approval was granted for outcome analysis in this study (WIRB Study#1275376; IRB#20200195; February 5, 2020), and informed consent was obtained for all patients for the relevant surgical procedures as well as anonymized inclusion into this study. All methods of this study were conducted following the relevant regulations for working with human subjects and the Declaration of Helsinki [14].

## Results

Patient demographics and baseline characteristics

The study analyzed 21 patients who underwent isolated CABG surgery, with seven patients developing postoperative AKI and 14 serving as controls. As seen in Table 2, demographic characteristics were well-matched between groups, with no significant differences in age (67.57 ± 9.95 vs 64.57 ± 12.22 years, p = 0.581) or gender distribution (85.71% male in both groups). Body mass index was comparable, though trending higher in the AKI group (30.43 ± 5.09 vs 28.07 ± 4.36 kg/m², p = 0.283). Preexisting cardiovascular risk factors were similarly distributed between groups. The prevalence of hypertension (71.43% vs 78.57%, p = 0.733), dyslipidemia (71.43% vs 78.57%, p = 0.733), and family history of coronary artery disease (42.86% in both groups, p > 0.999) was comparable. A history of previous myocardial infarction was observed in 28.57% of AKI patients compared to 50% of controls (p = 0.375), while previous percutaneous coronary intervention rates were 28.57% vs 42.86%, respectively (p = 0.549). Notably, while most preoperative characteristics showed no significant differences, preoperative NephroCheck values were significantly elevated in patients who subsequently developed AKI (1.34 ± 1.33 vs 0.54 ± 0.43, p = 0.009). Other renal function parameters including preoperative creatinine (0.90 ± 0.15 vs 0.90 ± 0.28 mg/dL, p = 0.847) and GFR (83.20 ± 16.85 vs 85.9 ± 28.63 mL/min/1.73m², p = 0.550) were similar between groups.

**Table 2:** Preoperative characteristics BMI: body mass index; CAD: coronary artery disease; COPD: chronic obstructive pulmonary disease; MI: myocardial infarction; PCI: percutaneous coronary intervention; CABG: coronary artery bypass grafting; STS: Society of Thoracic Surgeons; GFR: glomerular filtration rate; NIRS: near-infrared spectroscopy Categorical variables are represented as N (%) with parametric continuous variables being represented as mean ± SD and nonparametric continuous variables represented as median (IQR). Chi-square or Fisher exact tests were used for categorical variables, and T-tests or Mann-Whitney U tests were used for continuous variables

Variable	AKI (n = 7)	Control (n = 14)	p-value
Age (y)	67.57 ± 9.95	64.57 ± 12.22	0.581
Male (kg/m2)	6/7 (85.71)	12/14 (85.71)	>0.999
BMI	30.43 ± 5.09	28.07 ± 4.36	0.283
Family history of CAD	3/7 (42.86)	6/14 (42.86)	>0.999
Diabetes	1/7 (14.29)	3/14 (21.43)	0.712
COPD	1/7 (14.29)	0/14 (0.00)	0.163
Dyslipidemia	5/7 (71.43)	11/14 (78.57)	0.733
Hypertension	5/7 (71.43)	11/14 (78.57)	0.733
Tobacco use	3/7 (42.86)	6/14 (42.86)	> 0.999
Chronic lung disease	4/7 (57.14)	6/14 (42.86)	0.560
Immunosuppressed	3/7 (42.86)	3/14 (21.43)	0.330
Obesity	3/7 (42.86)	4/14 (28.47)	0.537
Alcohol use	1/7 (14.29)	1/14 (7.14)	0.621
Previous MI	2/7 (28.57)	7/14 (50.0)	0.375
Previous PCI	2/7 (28.57)	6/14 (42.86)	0.549
Prior CABG	0/7 (0.0)	1/14 (7.14)	0.494
Arrhythmia	1/7 (14.29)	2/14 (14.29)	> 0.999
Congestive heart failure	1/7 (14.29)	0/14 (0.0)	0.163
STS risk score	1.21 (0.52)	0.95 (0.53)	0.318
CHA2DS2VASC score	2.43 (0.79)	2.50 (1.99)	0.929
HASBLED score	2.29 (0.95)	2.36 (1.39)	0.905
Thakar score	0.4 (0.0)	0.5 (0.37)	0.494
Preoperative NephroCheck ((ng/ml)^2^/1000)	1.34 (1.33)	0.54 (0.43)	0.009
Preoperative creatinine (mg/dL)	0.90 (0.15)	0.90 (0.28)	0.847
Preoperative GFR (mL/min/1.73m²)	83.20 (16.85)	85.9 (28.63)	0.550
Preoperative right renal NIRS (%)	76.50 (13.44)	77.58 (11.63)	0.862
Preoperative left renal NIRS (%)	79.0 (6.69)	85.0 (6.52)	0.082

Intraoperative parameters and monitoring

Analysis of intraoperative characteristics revealed comparable surgical times and perfusion parameters between groups (Table 3). CPB duration (94.17 ± 33.39 vs 96.43 ± 24.36 minutes, p = 0.866) and aortic cross-clamp times (72.83 ± 16.73 vs 73.71 ± 19.14 minutes, p = 0.923) were similar. Intraoperative renal perfusion monitoring through NIRS showed comparable values for both left (79.67 ± 7.61 vs 81.50 ± 8.53, p = 0.663) and right (78.83 ± 14.58 vs 76.75 ± 11.68, p = 0.682) kidneys. While not reaching statistical significance, the AKI group demonstrated a trend toward higher net fluid balance (780 ± 645.76 vs 276.42 ± 728.22 mL, p = 0.201) despite lower urine output (557.14 ± 205.0 vs 632.14 ± 447.90 mL, p = 0.681).

**Table 3:** Intraoperative characteristics AKI: acute kidney injury; NIRS: near-infrared spectroscopy; IQR: interquartile range Categorical variables are represented as N (%) with nonparametric continuous variables represented as median (IQR). Chi-square or Fisher exact tests were used for categorical variables, and T-tests or Mann-Whitney U tests were used for continuous variables

	Variable	AKI (n = 7)	Control (n = 14)		p-value
Intraoperative right renal NIRS (%)		78.83 (14.58)	76.75 (11.68)	0.682	
Intraoperative left renal NIRS (%)		79.67 (7.61)	81.50 (8.53)	0.663	
Postoperative right renal NIRS (%)		76.67 (14.31)	64.88 (24.35)	0.427	
Postoperative left renal NIRS (%)		77.67 (6.38)	78.50 (6.40)	0.798	
Urine output (mL)		557.14 (205.0)	632.14 (447.90)	0.681	
Net volume (mL)		780 (645.76)	276.42 (728.22)	0.201	
Cardiopulmonary bypass time (min)		94.17 (33.39)	96.43 (24.36)	0.866	
Cross-clamp time (min)		72.83 (16.73)	73.71 (19.14)	0.923	

Postoperative course and outcomes

Postoperative outcomes analysis showed comparable hospital courses between groups (Table 4). The total length of hospital admission (5.43 ± 1.40 vs 5.0 ± 1.49 days, p = 0.364) and ICU stay duration (4.0 ± 1.63 vs 3.4 ± 1.07 days, p = 0.260) were similar. Vasopressor support requirements were also comparable, with 57.14% of AKI patients requiring vasopressin compared to 42.86% of controls (p = 0.434). When vasopressor support was needed, the duration was similar between groups (1.75 ± 0.50 vs 1.67 ± 0.52 days, p = 0.807). CVICU admission NephroCheck values showed a trend toward being higher in the AKI group (0.49 ± 0.59 vs 0.09 ± 0.04, p = 0.064), though not reaching statistical significance. One patient in the AKI group required temporary renal replacement therapy for two days (dialysis).

**Table 4:** Postoperative outcomes CVICU: cardiovascular intensive care unit; ICU: intensive care unit; AKI: acute kidney injury Categorical variables are represented as N (%) with nonparametric continuous variables represented as median (IQR). Chi-square or Fisher exact tests were used for categorical variables, and Mann-Whitney U tests were used for continuous variables

Variable	AKI (n = 7)	Control (n = 14)	p-value
CVICU admit NephroCheck ((ng/ml)^2^/1000)	0.49 (0.59)	0.09 (0.04)	0.064
Length of stay (days)	5.43 (1.40)	5.0 (1.49)	0.364
ICU stay (days)	4.0 (1.63)	3.4 (1.07)	0.260
Vasopressin	4/7 (57.14)	6/14 (42.86)	0.434
Days on vasopressin	1.75 (0.50)	1.67 (0.52)	0.807

Predictors of AKI

Regression analysis was performed to identify variables predictive of postoperative AKI development (Table 5). Preoperative NephroCheck emerged as the only significant predictor, with an odds ratio of 17.85 (p = 0.047). Traditional risk factors including preoperative renal function parameters (GFR: OR 0.99, p = 0.682; creatinine: OR 1.00, p = 1.000), STS score (OR 2.56, p = 0.304), and operative times (CPB time: OR 1.00, p = 0.905; cross-clamp time: OR 1.01, p = 0.846) did not show significant predictive value. Similarly, intraoperative renal NIRS monitoring was not predictive of AKI development (left: OR 0.99, p = 0.920; right: OR 1.02, p = 0.650).

**Table 5:** Regression analysis to identify predictors of postoperative acute kidney injury FPR: false positive rate; FNR: false negative rate

Variable	Odds ratio	p-value	Sensitivity	Specificity	FPR	FNR
Aortic cross-clamp time	1.01	0.8463	0.00	1.00	0.00	1.00
Cardiopulmonary bypass time	1.00	0.9045	0.00	1.00	0.00	1.00
Intraoperative renal NIRS: left	0.99	0.9201	0.00	1.00	0.00	1.00
Intraoperative renal NIRS: right	1.02	0.6504	0.00	1.00	0.00	1.00
Preoperative creatinine	1.00	1.0000	0.00	1.00	0.00	1.00
Preoperative GFR	0.99	0.6815	0.00	1.00	0.00	1.00
Preoperative NephroCheck	17.85	0.0471	0.71	0.93	0.07	0.29
Preoperative renal NIRS: Left	0.86	0.1163	0.43	0.93	0.07	0.57
Preoperative renal NIRS: Right	0.99	0.8048	0.00	1.00	0.00	1.00
Preoperative urine output	1.00	0.2040	0.00	0.86	0.14	1.00
STS score	2.56	0.3038	0.14	0.86	0.14	0.86

## Discussion

The findings of this study underscore the utility of the NephroCheck test in the perioperative management of patients undergoing cardiac surgery. By assessing preoperative fluid status, the NephroCheck test enabled more precise intraoperative fluid resuscitation, particularly in patients identified as being very dry. This targeted approach to fluid management was instrumental in mitigating the risk of postoperative renal failure.

The ability to identify patients at risk of dehydration preoperatively allowed for tailored intraoperative interventions. Specifically, when the NephroCheck test indicated a high risk of AKI due to low fluid status, clinicians could administer fluids more judiciously during surgery. This proactive strategy not only optimized hemodynamic stability but also reduced the incidence of CSA-AKI, highlighting the importance of personalized fluid management in improving surgical outcomes. This aligns with previous research indicating that maintenance of renal perfusion and balanced fluid management are critical in preventing CSA-AKI [15,16].

Moreover, the dual application of the NephroCheck test both preoperatively and intraoperatively proved to be beneficial. Preoperative assessments provided a baseline fluid status, while intraoperative monitoring facilitated real-time adjustments to fluid therapy. This dynamic approach ensured that patients received the appropriate volume of fluids, thereby preventing the detrimental effects of both under-resuscitation and over-resuscitation. Studies have shown that biomarkers like IGFBP7 and TIMP2 are effective in the early detection of kidney stress, allowing for timely interventions [17,18].

The implications of these findings are significant. By incorporating NephroCheck into routine preoperative assessments, healthcare providers can enhance patient care through timely and personalized interventions. This not only has the potential to reduce the incidence of CSA-AKI but also to alleviate the associated clinical and economic burdens. The reduction in postoperative renal failure translates to shorter hospital stays, decreased need for renal replacement therapy, and overall improved patient outcomes. The economic impact of CSA-AKI is substantial, with hospitalization costs nearly doubling for affected patients [19].

Limitations

This study has several notable limitations. The small sample size of only 21 patients, with just seven developing AKI, significantly limits statistical power and generalizability. Its single-center retrospective design introduces potential selection bias and makes it difficult to establish causation. The study faced protocol inconsistencies, requiring two different testing timelines due to missing values and staffing issues. The follow-up period was limited to just seven postoperative days, potentially missing later AKI developments. By excluding patients with CKD, heart failure, and elevated creatinine, the findings may not apply to higher-risk populations. Despite discussing economic implications, no cost-effectiveness analysis was performed. The missing NephroCheck measurements that necessitated protocol changes suggest data collection problems. The study also lacks standardized intervention protocols based on NephroCheck values and does not provide long-term outcome data beyond hospital discharge. As acknowledged in their conclusion, larger prospective studies are needed to validate these findings and establish standardized protocols.

In conclusion, the NephroCheck test serves as a valuable tool in the perioperative management of cardiac surgery patients. Its ability to guide fluid resuscitation based on preoperative and intraoperative assessments can significantly mitigate the risk of postoperative renal failure. Future studies should continue to explore the broader applications of NephroCheck in various surgical contexts to further validate its efficacy and optimize patient care protocols.

## Conclusions

This study demonstrates that preoperative NephroCheck testing is a significant predictor of cardiac surgery-associated AKI following CABG, with elevated preoperative values showing a strong correlation with postoperative AKI development (OR 17.85, p = 0.047). Notably, NephroCheck outperformed traditional risk assessment methods, including preoperative renal function parameters, STS scores, and operative times, none of which showed significant predictive value. The study's findings suggest that incorporating NephroCheck into standard preoperative protocols could enable more targeted preventive interventions through optimized fluid management and perioperative care strategies. This could potentially reduce CSA-AKI incidence and its associated complications, though larger prospective studies are needed to further validate these findings and establish standardized intervention protocols based on NephroCheck values. Future research should focus on determining optimal cutoff values for risk stratification and evaluating the cost-effectiveness of routine NephroCheck screening in cardiac surgery patients.
